# AMS Radiocarbon Dating of Large Za Baobabs (*Adansonia za*) of Madagascar

**DOI:** 10.1371/journal.pone.0146977

**Published:** 2016-01-13

**Authors:** Adrian Patrut, Roxana T. Patrut, Pascal Danthu, Jean-Michel Leong Pock-Tsy, Laszlo Rakosy, Daniel A. Lowy, Karl F. von Reden

**Affiliations:** 1 Babes-Bolyai University, Faculty of Chemistry, Cluj-Napoca, Romania; 2 Babes-Bolyai University, Faculty of Biology and Geology, Cluj-Napoca, Romania; 3 Cirad, UPR BSEF, Montpellier, France; 4 Direction régionale du CIRAD, Antananarivo, Madagascar; 5 DP Forêt et Biodiversité, Antananarivo, Madagascar; 6 Nova University, Alexandria Campus, Alexandria, Virigina, United States of America; 7 NOSAMS Facility, Dept. of Geology&Geophysics, Woods Hole Oceanographic Institution, Woods Hole, Massachusetts, United States of America; New York State Museum, UNITED STATES

## Abstract

The article reports the radiocarbon investigation of Anzapalivoro, the largest za baobab (*Adansonia za*) specimen of Madagascar and of another za, namely the Big cistern baobab. Several wood samples collected from the large inner cavity and from the outer part/exterior of the tree were investigated by AMS (accelerator mass spectrometry) radiocarbon dating. For samples collected from the cavity walls, the age values increase with the distance into the wood up to a point of maximum age, after which the values decrease toward the outer part. This anomaly of age sequences indicates that the inner cavity of Anzapalivoro is a false cavity, practically an empty space between several fused stems disposed in a ring-shaped structure. The radiocarbon date of the oldest sample was 780 ± 30 bp, which corresponds to a calibrated age of around 735 yr. Dating results indicate that Anzapalivoro has a closed ring-shaped structure, which consists of 5 fused stems that close a false cavity. The oldest part of the biggest za baobab has a calculated age of 900 years. We also disclose results of the investigation of a second za baobab, the Big cistern baobab, which was hollowed out for water storage. This specimen, which consists of 4 fused stems, was found to be around 260 years old.

## Introduction

The genus *Adansonia* belonging to the Bombacoideae, a subfamily of Malvaceae, consists of nine species. Six species are endemic to Madagascar and have a natural distribution only on this island [1–4].

In 2005, we started in-depth research for elucidating several controversial or less understood aspects concerning the African baobab (*Adansonia digitata* L.). This research is based on our new approach, which enables the dating of standing and live specimens by AMS radiocarbon investigation of small wood samples collected from their inner cavities and/or from different areas of their trunk/stems. Due to the peculiar ability of baobabs to produce stems periodically during their life cycle, they develop over time structures of increasing complexity. Therefore, we focused on the investigation of superlative individuals, *i*.*e*., very large and potentially old baobabs. One should mention that neither the identification of such very complex architectures nor the accurate dating of old baobabs are possible by means of the traditional dendrochronological methods, which are based on tree ring investigation.

The radiocarbon investigation of large African baobabs has demonstrated that their architecture is much more complex than previously believed. Our approach also involves a very careful analysis and interpretation of the AMS radiocarbon dating results. The obtained results have demonstrated that all large baobabs are multi-stemmed. We identified the so-called open and closed ring-shaped structures, which are the most important architectures that enable African baobabs to reach old ages and large sizes. We also identified the false cavities, which are large natural empty spaces between fused stems disposed in a closed ring-shaped structure. The oldest dated *A*. *digitata* were found to have ages up to 2,000 yr. According to these values, the African baobab becomes the angiosperm with the longest life span [5–11].

On the other hand, dated tree rings of several studied baobab specimens, which may act as a proxy climate archive, were used for past climate reconstruction in Southern Africa [12, 13].

We extended our research on the architecture, growth and age of the *Adansonia* genus by starting to investigate, by using the same approach based on AMS radiocarbon dating [7, 9], large individuals of the most representative three Malagasy species, namely *Adansonia rubrostipa* Jum. & H. Perrier (fony baobab), *Adansonia za* Baill. (za baobab) and *Adansonia grandidieri* Baill. (Grandidier’s baobab or Reniala) [1–3, 14, 15]. Another aim of our research is to identify the oldest baobab specimen of Madagascar.

Up to the present, we published the AMS radiocarbon dating results of large and old Malagasy baobabs which belong to *A*. *rubrostipa* [16] and *A*. *grandidieri* [17]. We found that big individuals of both species may reach ages well over 1000 years.

**Fig 1:**
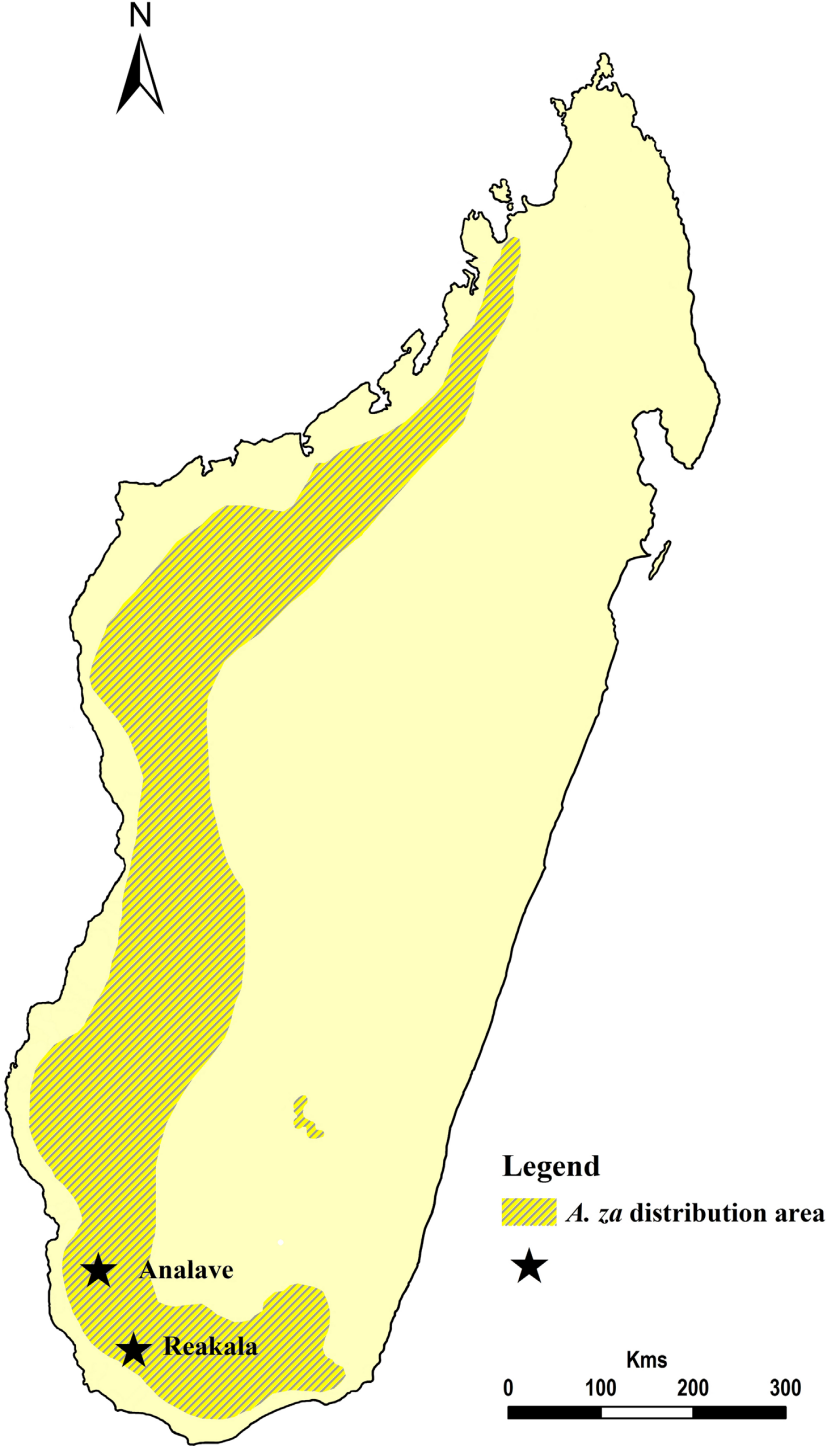
Map of Madagascar showing the large distribution area of *Adansonia za* and the location of the two investigated baobabs, Reakala and Analave in the southern Toliara province.

Due to its great ecological plasticity, *A*. *za* is the most widespread baobab species of Madagascar and can be found in areas with very different climate conditions. It is endemic to Northwestern and particularly to Southern Madagascar (Fig 1). According to latest estimates, the total population of *A*. *za*, which covers a large area of distribution, is around 3 million mature individuals.

In terms of general description of the species, *A*. *za* is represented by short (10 m) to tall (20–25 m) deciduous trees, usually with single, cylindrical or slightly tapering trunks, often with irregular swellings. The crown is rounded and the primary branches are typically ascending and tapering. The trunk and branches have a grey or a brownish-rose colour [1–3]. Similarly to *A*. *grandidieri*, the height, girth and shape of mature and old *A*. *za* specimens may be very different and usually depend on their locations.

Besides their ecological role, especially in stabilising dry forest ecosystems, the Malagasy baobabs are multipurpose trees and have numerous functions and uses. Their fruits, seeds, leaves, bark and wood are greatly appreciated by the rural communities [3, 18, 19]. In areas with a long dry season of Southern Madagascar, *A*. *za* trees also have a very special use. The trunks of some *A*. *za* are hollowed out and used as tanks for water storage. These trees, called “cistern baobabs”, are located on the calcareous Mahafaly plateau, in the area of the small town Ampanihy, more precisely at Analave, Ampotaka and Andremba. In the very dry Mahafaly plateau area, the water sources are extremely sporadic and the few raindrops evaporate quickly in the lime soil. That is why the water storage is vital for the local population. The hollows dug in the porous trunks of za baobabs act like water reservoirs. The hollows are filled with rainwater during the rainy season (December-March); the stored water is used in households over the long dry season (April-November). Each cistern baobab belongs to a tribal family of the Mahafaly or Tanàla communities and is used exclusively by its membres. The total number of cistern baobabs is up to 1000.

We investigated representative *A*. *za* specimens for determining their architecture, growth and age. Here we disclose results of the AMS radiocarbon investigation of Anzapalivoro, the biggest za baobab and also of the largest cistern baobab of Analave.

## Materials and Methods

### Ethical statement

The investigation and sampling of the baobabs was authorised by the Forestry Direction of the Ministry of Environment, Ecology and Forestry of Madagascar and by the Madagascar National Parks. The sampling of Anzapalivoro was also approved by the local community of Reakala. The Madagascar National Parks and PARRUR project (ECOBAO) provided scientific assistance and support in the investigation. After each coring, the increment borer was disinfected with methyl alcohol. The small coring holes were sealed with Steriseal (Efekto), a special polymer sealing product, for preventing any possible infection of the trees.

### The baobabs and their area

**Fig 2:**
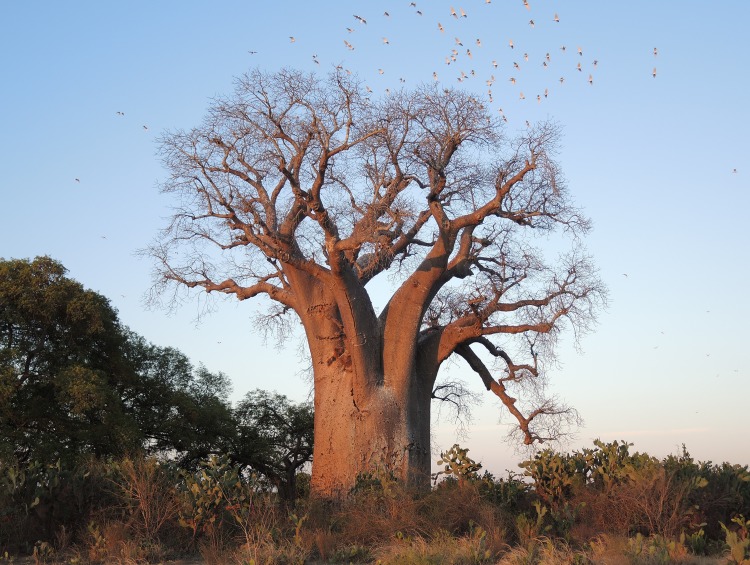
General view of the spectacular Anzapalivoro (“the sacred za baobab, palace of birds”), which is surrounded by a flock of white birds.

**Fig 3:**
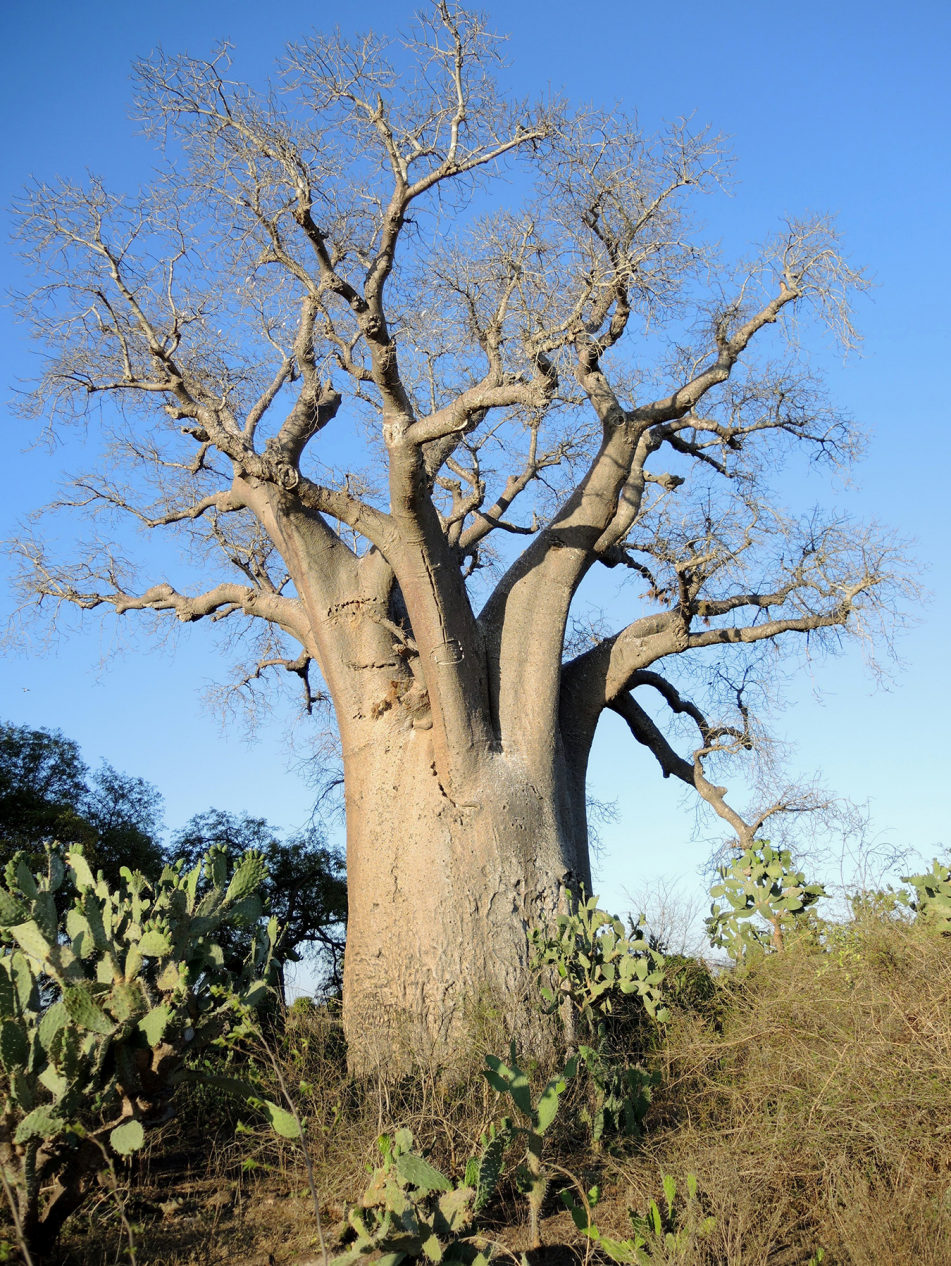
Another image of Anzapalivoro, which rises from a field of cacti, showing its big trunk and the large branches.

Anzapalivoro (in Mahafaly, *i*.*e*.,”the sacred za, palace of birds”), also called the “big za of Ampanihy”, is located close to the village Reakala, at 23 km north-west of Ampanihy, in the Toliara province, Madagascar (Fig 2). Its GPS coordinates are 24°40.688' S, 044°33.725' E and the altitude is 259 m. The mean annual rainfall in the area is 387 mm (Ampanihy). Anzapalivoro, which is by far the biggest za baobab, has a height of 26.3 m and the circumference at breast height (cbh; *i*.*e*., at 1.30 m) is 22.25 m. The big quasi-cylindrical trunk divides at heights between 7.5–11.9 m into 8 large primary branches (Fig 3). The canopy has an extension of 29.5 (N-S) x 32.2 m (W-E). The bark, which covers the trunk and branches, has a grey colour, with reddish-brown reflexes at sunset. According to measurements, the total volume of Anzapalivoro (including the inner cavity) is 330 m^3^, out of which 285 m^3^ belong to the trunk and 45 m^3^ to the canopy. By this volume value, Anzapalivoro is the third biggest baobab of Madagascar, after the two very large *A*. *grandidieri* individuals of Andombiro [17], and also one of the 10 largest *Adansonia* specimens of the world.

**Fig 4:**
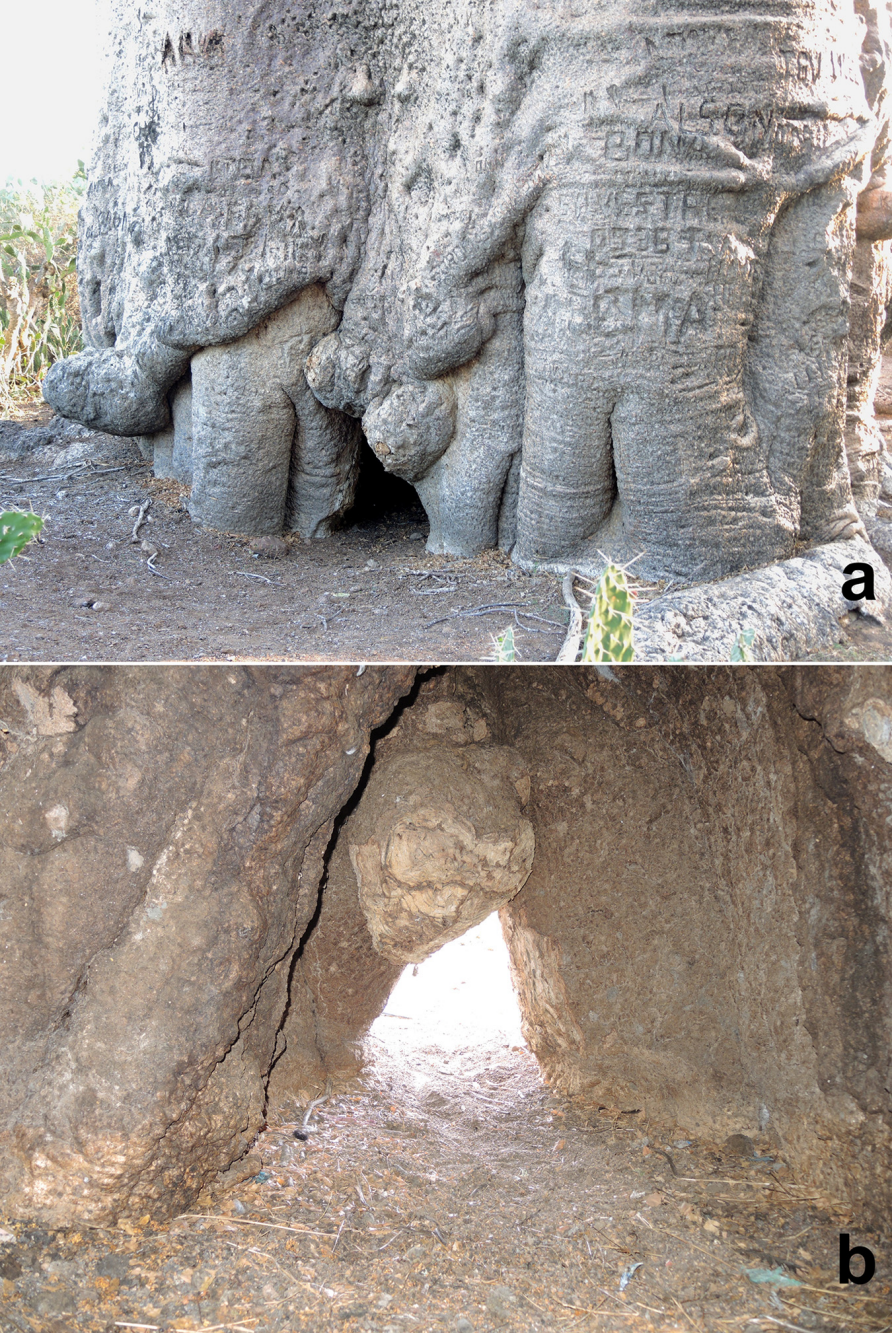
The entrance to the inner cavity of Anzapalivoro, photographed from the exterior (a) and from the interior (b).

The big za possesses a large inner cavity, which is accessible *via* a long corridor. The small triangular entrance to the corridor (base 0.70 m, height 0.65 m) is located at the base of the northern flank of the tree (Fig 4). The corridor has a total length of 2.30 m, its width increases from 0.70 to 1.60 m and the height from 0.65 to 2.10 m. The mushroom-like shaped actual cavity has the following dimensions: 2.65 m (N-S) x 3.60 m (W-E) at ground level and 2.68 m (N-S) x 3.45 m (W-E) at 1.30 m above ground. The total basal surface of the inner cavity (8.3 m^2^), including the corridor (2.7 m^2^), is 11.0 m^2^. The cavity, with its walls completely covered by bark, has also several high openings and several hundreds of bats live inside. By its height of 9.75 m, the cavity of Anzapalivoro is the tallest known for any individual belonging to the *Adansonia* species. The total volume of the inner cavity of the big za, namely 54 m^3^, is the third largest measured for a baobab, after the false cavity of the baobab of Samba Dia (70 m^3^) and the false double cavity of the Platland tree (65 m^3^), two large *A*. *digitata* specimens of Senegal and South Africa [9]. Anzapalivoro, which rises completely isolated from the middle of a cactus field, is certainly one of the most spectacular baobabs of the world. One should also stress that Anzapalivoro is at least twice as big (in terms of wood volume) and, likely also considerably older than any other za baobab. This is probably due to intense deforestation and the slash-and-burn farming method used by the Malagasy populations.

The big cistern baobab is the largest *A*. *za* used for water storage. It is located close to the village of Analave, at 30 km south-east of Betioky, in the Toliara province. Its GPS coordinates are 23°53.215' S, 044°11.267' E and the altitude is 244 m. The mean annual rainfall in the area is 402 mm (Betioky), but almost half of this amount may fall in only 1–2 days.

**Fig 5:**
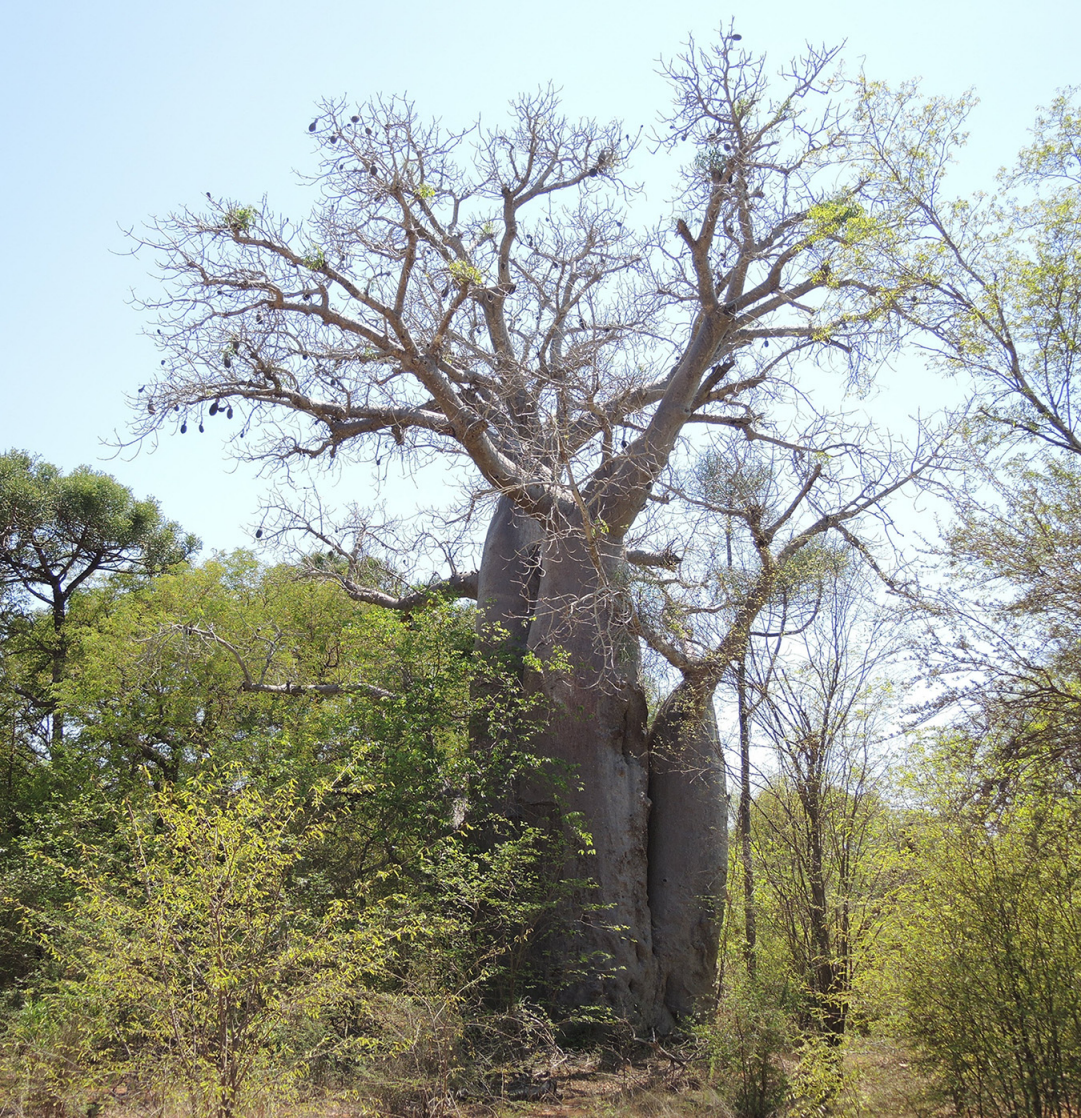
General view of the Big cistern za with its 4 partially fused stems.

**Fig 6:**
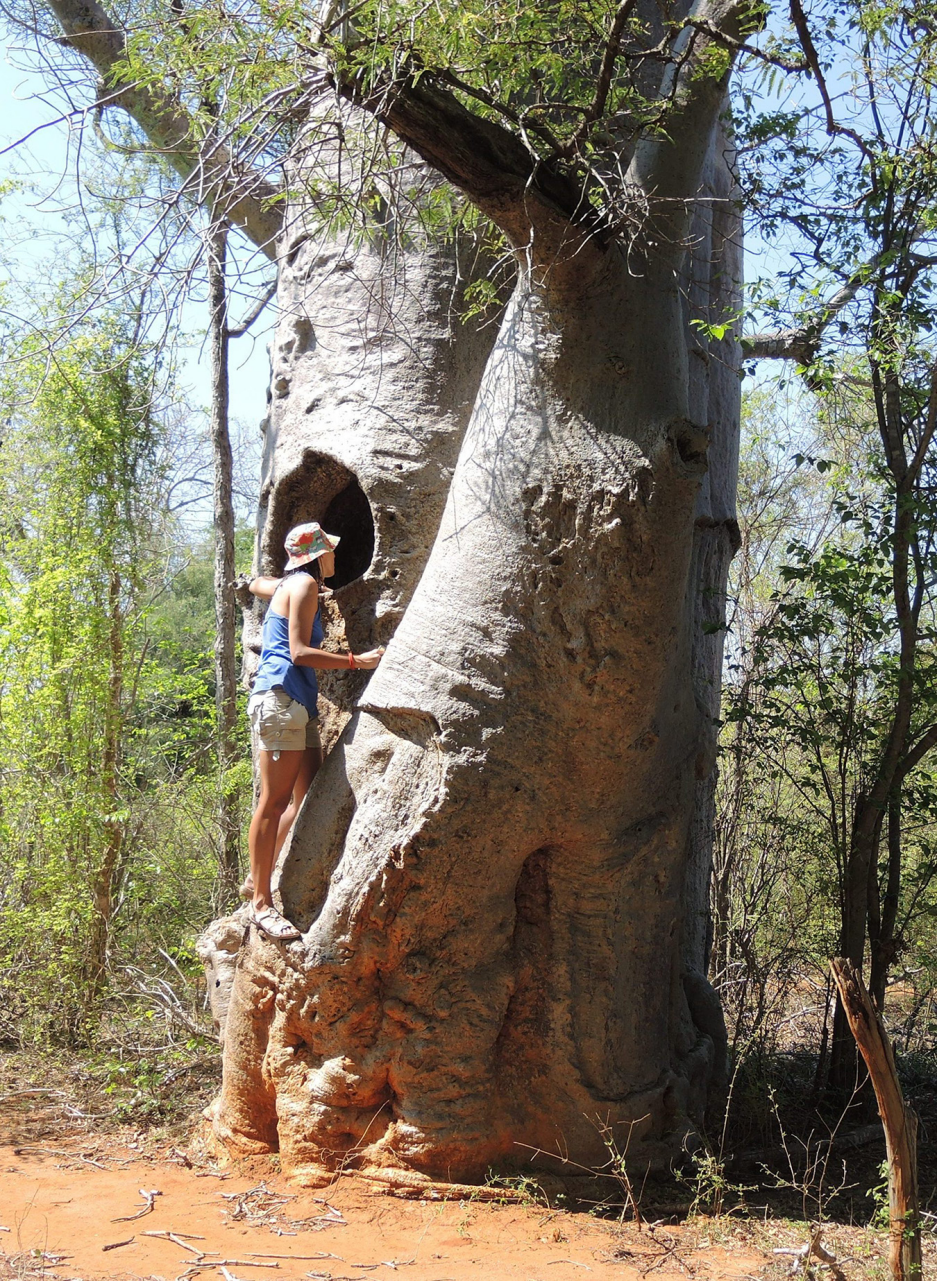
The image presents the high opening (2.60 m) to the cistern which was dug in the largest stem.

The tree has a height of 16.2 m and the circumference at breast height (cbh) is 12.10 m. Its trunk consists of 4 partially fused stems of different dimensions, which form a cluster structure (Fig 5). The largest stem was hollowed out at the height of 2.60 m from the ground (Fig 6). The quasi-circular hole has a diametre which varies between 0.60 and 0.70 m and leads to the cylindrical hollow inside the stem. This hollow, which is used for water storage during the dry season has an inner diametre of 0.60 m and a height of 1.58 m; its total volume of 0.447 m^3^ can stock up to ca. 450 L rainwater.

### Sample collection

**Fig 7:**
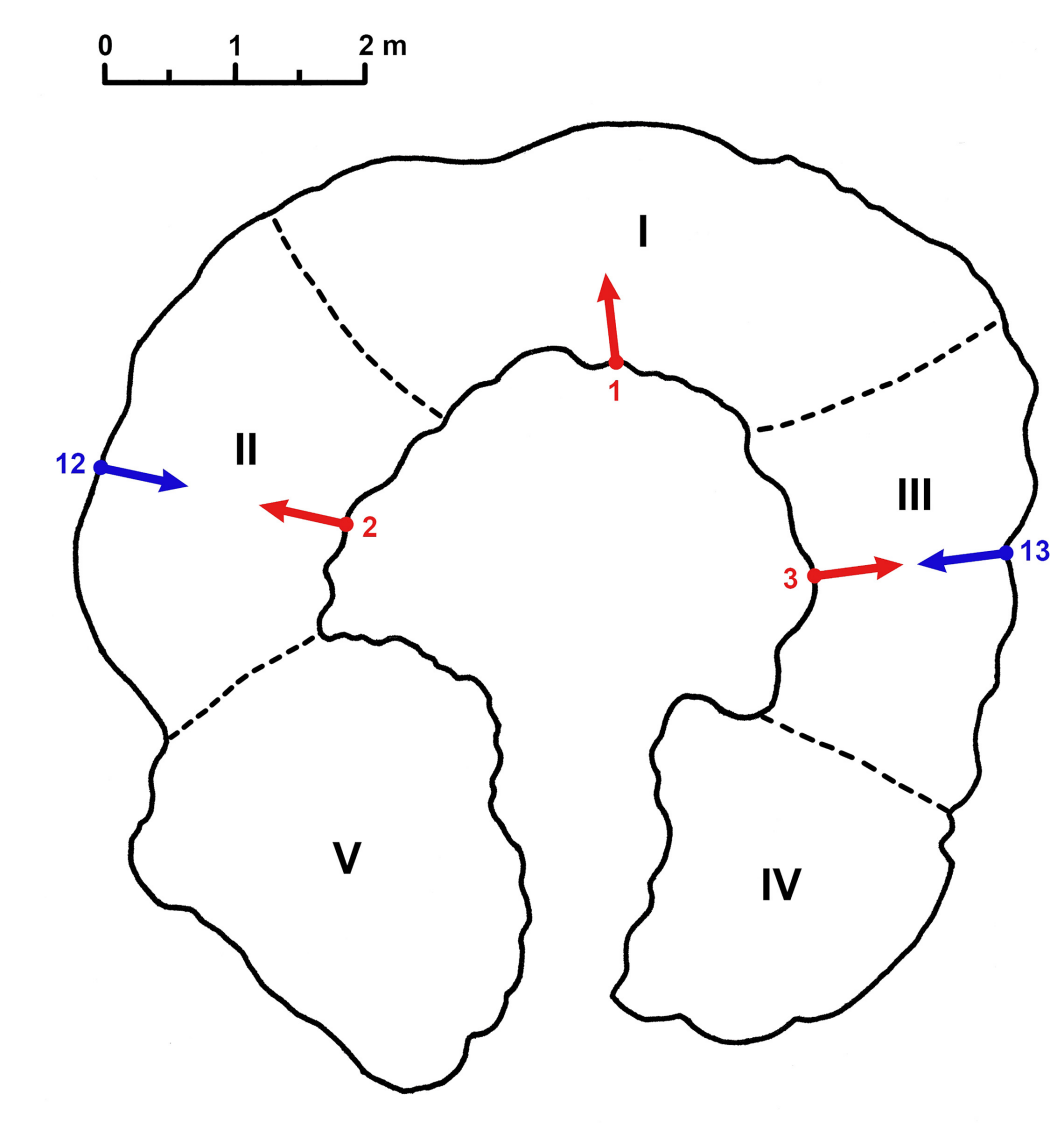
Cross-section of Anzapalivoro (at 1 m above ground), displaying the false cavity, the projection of the positions of the three internal sampling points (labelled 1, 2 and 3) and of the two external sampling points (labelled 12 and 13), with the sampling directions. The five fused stems (labelled I, II, III, IV and V) are also shown.

We collected 5 wood samples from Anzapalivoro as follows: 3 samples (labelled AZA-1, AZA-2 and AZA-3) were collected from the inner cavity walls, at convenient heights between 1.27–1.53 m, while 2 samples (labelled AZA-12 and AZA-13) were collected from the outer part/exterior of the trunk, at heights of 1.44–1.47 m. One should stress that samples AZA-2 and AZA-12, as well as AZA-3 and AZA-13 are opposite, *i*.*e*., they were collected from opposite directions (inner cavity *vs*. outer part/exterior of the trunk). The sampling positions are shown in Fig 7. Another sample was collected from the hollow carved in the big cistern za (labelled BCZ-1), at the height of 2.70 m.

**Fig 8:**
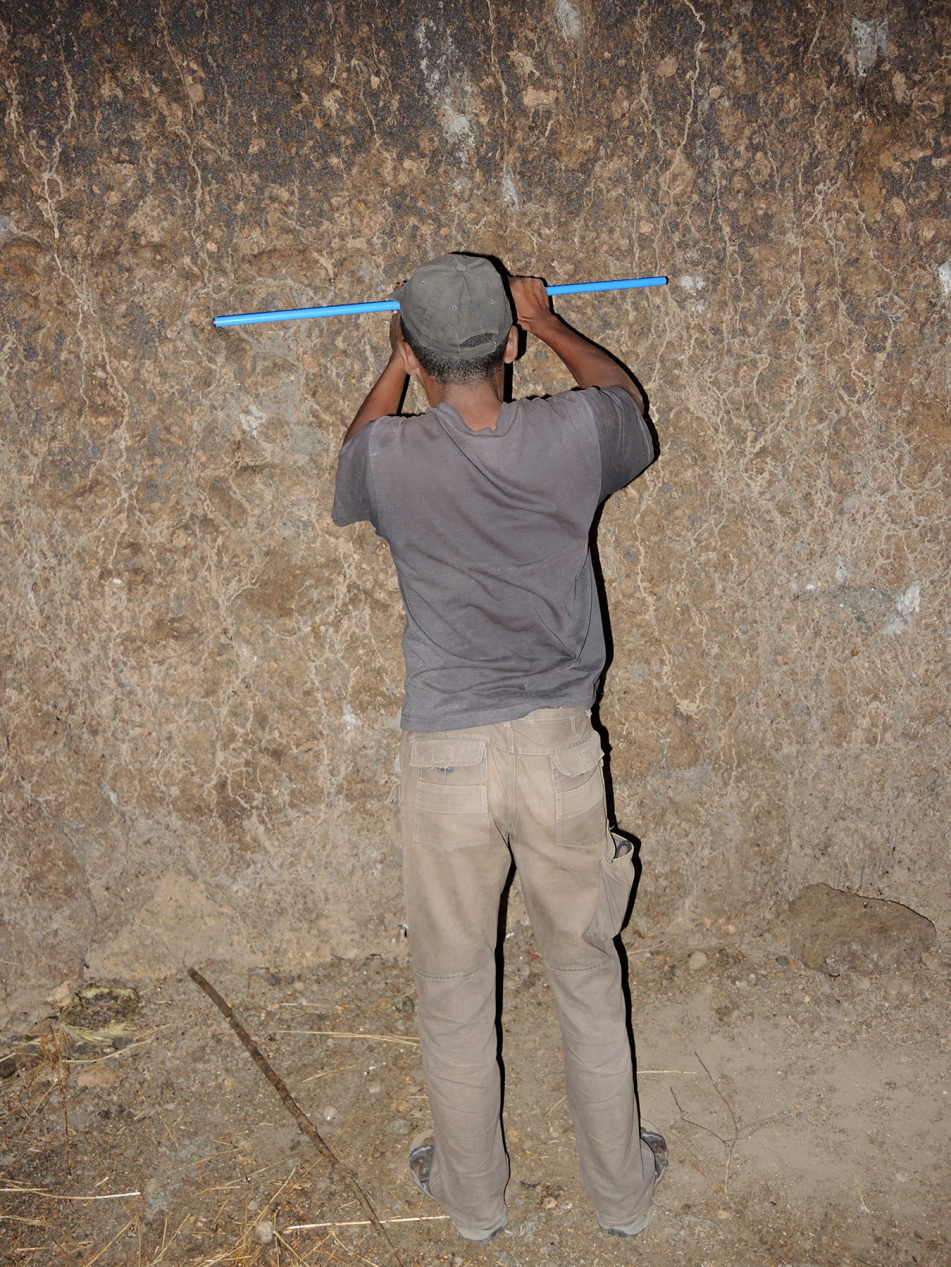
Collecting samples from the false inner cavity of Anzapalivoro. One can notice the bark which covers the cavity walls.

The samples were collected by using Haglöf increment borers (0.80–1.00 m long, 0.012 m inner diametre), see Fig 8. Twelve tiny pieces/segments were extracted from determined positions of the original samples. These segments (also labelled a, b, c, d, and e) were processed and investigated by AMS radiocarbon dating.

### Sample preparation

The standard acid-base-acid pretreatment method [20, 6] was used for removing soluble and mobile organic components. The pretreated sample segments were combusted to CO_2_ *via* the closed tube combustion method [21]. Next, CO_2_ was reduced to graphite on iron catalyst, under hydrogen atmosphere [22]. The resulting graphite samples were analysed by AMS.

### AMS measurements

AMS radiocarbon measurements were performed at the NOSAMS Facility of the Woods Hole Oceanographic Institution by using the Pelletron®Tandem 500 kV AMS system [23, 24]. The obtained fraction modern values corrected for isotope fractionation were ultimately converted to a radiocarbon date.

### Calibration

Radiocarbon dates were calibrated and converted into calendar ages with the OxCal v4.2 for Windows [25], by using the SHCal13 atmospheric data set [26].

## Results

### AMS results and calibrated ages

Radiocarbon dates of the 12 samples/segments which originate from Anzapalivoro (AZA) and Big cistern za (BCZ) are displayed in Table 1. Radiocarbon dates and errors were rounded to the nearest year. The 1σ probability distribution (68.2%) was selected to derive calibrated age ranges. For 2 sample segments, the 1σ distribution is consistent with one range of calendar years, while for other 7 segments the 1σ distribution corresponds to two or three ranges. For these segments, the confidence interval of one range is considerably greater than that of the others and this range is consistent with the age sequence of the corresponding sample and the estimated growth rates; therefore, it was selected as the cal ad range of the sample for the purpose of this discussion. For 3 sample segments with the lowest radiocarbon dates, the calibration leads to four, five and even six 1σ calendar age ranges for each date. In these cases, we selected the range with the highest probability as the cal ad range for each segment; one should mention that the calibration of such low radiocarbon dates is always difficult.

**Table 1:** AMS radiocarbon dating results and calibrated calendar ages of samples collected from Anzapalivoro (AZA) and Big cistern za (BCZ).

Sample/ segment code	Depth^1^ (10^−2^ m)	Radiocarbon date [error] (^14^C yr bp)	Cal ad range 1σ [confidence interval]	Sample/segment age [error] (yr)	Accession #
AZA-1a	31	440 [± 20]	**1450–1486 [68.2%]**	545 [± 20]	OS-97144
AZA-2a	34	340 [± 24]	**1510–1576 [56.0%]** 1622–1636 [12.2%]	470 [± 35]	OS-95070
AZA-2b	51	780 [± 30]	1233–1244 [12.8%] **1264–1292 [55.4%]**	735 [±15]	OS-98676
AZA-3a	32	310 [± 23]	1515–1542 [23.3%] **1625–1654 [44.9%]**	375 [± 15]	OS-97145
AZA-3b	42	353 [± 25]	**1508–1584 [61.4%]** 1620–1628 [6.8%]	465 [± 40]	OS-109521
AZA-3c	52	557 [± 19]	**1409–1429 [68.2%]**	595 [± 10]	OS-110220
AZA-3d	62	335 [± 25]	**1510–1575 [51.5%]** 1622–1640 [16.7%]	470 [± 30]	OS-91358
AZA-3e	72	190 [± 20]	**1672–1698 [23.8%]** 1724–1745 [17.2%] 1758–1780 [13.4%] 1796–1808 [10.4%] 1870–1876 [3.5%]	330 [± 15]	OS-109281
AZA-12a	77	280 [± 25]	**1632–1669 [63.7%]** 1786–1792 [4.5%]	360 [± 25]	OS-90745
AZA-13a	25	127 [± 22]	1710–1720 [6.9%] 1812–1836 [18.6%] 1847–1857 [6.2%] **1880–1928 [36.4%]**	110 [±25]	OS-108122
AZA-13b	72	180 [± 20]	**1674–1707 [23.5%]** 1720–1740 [12.2%] 1797–1811 [9.5%] 1837–1846 [4.9%] 1858–1879 [9.5%] 1930–1950 [8.6%]	325 [± 15]	OS-90684
BCZ-1	20	232 [± 20]	1664–1672 [9.8%] **1742–1770 [33.5%]** 1778–1796 [24.9%]	260 [± 15]	OS-119877

^1^ Depth in the wood from the sampling point.

For obtaining single age values, we derived a mean cal age of each segment from the selected range (marked in bold). Sample/segment ages represent the difference between ad 2015 and the mean value of the selected range, with the corresponding error. Ages and errors were rounded to the nearest 5 years.

### Dating results of samples

In the case of Anzapalivoro, we dated 11 segments, out of which 5 segments (from AZA-3a to AZA-3e) were extracted from the longest cavity sample AZA-3, while 2 segments (AZA-13a and AZA-13b) originate from the opposite outer sample AZA-13. Two segments (AZA-2a and AZA-2b) were also extracted from the cavity sample AZA-2.

With two exceptions (AZA-3d and AZA-3e), radiocarbon dates of segments increase with the distance from the sampling point, *i*.*e*., with the depth into the wood. These exceptions are very significant and will be discussed later. The oldest segments of the 3 samples are AZA-2b, AZA-3c and AZA-13b, located at depths of 0.51, 0.52 and 0.72 m from the sampling points. Their radiocarbon dates of 780 ± 30 bp, 557 ± 19 bp and 180 ± 20 bp (before present, *i*.*e*., before the reference year ad 1950) correspond to calibrated ages of 735 ± 15, 595 ± 10 and 325 ± 15 yr.

The other 2 dated segments are practically the deepest/innermost ends of the cavity samples AZA-1 and from the outer sample AZA-12, labelled AZA-1a and AZA-12a, which originate from depths of 0.31 and 0.77 m from the corresponding sampling points. Their radiocarbon dates of 440 ± 20 bp and 280 ± 25 bp correspond to calibrated ages of 545 ± 20 and 360 ± 25 yr.

We also dated a segment close to the pith of the largest stem of the Big cistern za (BCZ). Its radiocarbon date of 232 ± 20 bp corresponds to a calibrated age of 260 ± 15 yr.

## Discussion

### Anomalous age sequence of cavity samples

Many old baobabs have large hollows, especially in the central area of their trunk/stems. In such large normal cavities, which are formed by wood removal, the pith/centre of the stem is located inside the cavity. Consequently, for samples collected from normal cavities, ages should decrease continuously from the cavity walls toward the outer part of the stem [1, 7, 9, 11, 27, 28].

Nevertheless, for samples collected from the inner cavity of Anzapalivoro, namely AZA-2 and AZA-3, we found that ages of dated segments increase with the depth in the wood. Furthermore, for the longest cavity sample AZA-3, ages increase from the cavity walls up to a certain distance into the wood (located at around 0.52 m from the sampling point), after which the values decrease toward the outer part, see Table 1.

We identified for the first time the same major anomaly of the age sequences of cavity samples dated by radiocarbon in our research of large and old *A*. *digitata* specimens. The only reasonable explanation for this finding is that such cavities are in fact natural empty spaces, which were never filled with wood, between several fused stems disposed in a closed ring-shaped structure. We named them false cavities [11, 28]. In all these cases, the age sequence of samples collected from inner cavities exhibits a continuous increase from the cavity walls up to a certain distance into the wood, which corresponds to an area of maximum age. The point/area of maximum age, which is the oldest part of the stem, is typically hollow as a result of decay. The oldest part of the fused stems is located between the false cavity walls and the outer part/exterior of each stem, always closer to the cavity. In a very limited number of cases, by using long increment borers, we were able to penetrate from the cavity walls deeper in the wood than the point of maximum age; in this area, the age values decrease continuously toward the exterior [11, 28].

The cavity sample AZA-3, which has a continuous length of 0.72 m and from which we dated 5 segments, is a very special case (Table 1). The ages increase from the cavity wall/sampling point (0 m), *via* the segments AZA-3a (0.32 m; 375 yr) and AZA-3b (0.42 m; 465 yr) up to AZA-3c (0.52 m; 595 yr), after which they decrease toward the exterior *via* the segments AZA-2d (0.62 m; 470 yr) and AZA-2e (0.72 m; 330 yr). The opposite sample AZA-13, which was collected from the outer part in the opposite direction, has also a continuous length of 0.72 m. As expected, the age values increase from the outer bark (0 m) with the distance into the wood, *via* the segments AZA-13a (0.25 m, 110 yr) and AZA-13b (0.72 m; 325 yr). The distance between the cavity wall and the outer part, *i*.*e*., between the opposite sampling points AZA-3 and AZA-13, is 1.45 m. This distance is practically covered by the cumulated length of samples AZA-3 and AZA-13, which is 0.72 + 0.72 = 1.44 m. In this special case, segments extracted from two samples, collected in opposite directions, allow to establish a complete age sequence from the cavity wall to the corresponding outer part of the stem in forward and backward directions, see Table 2. The point of maximum age is located at ca. 0.52 m from the inner cavity wall and ca. 0.93 m from the outer part of the stem. One should also mention that segments AZA-3e and AZA-13b, which are only 0.01 m away from another, have almost identical ages, namely 330 *vs*. 325 yr.

**Table 2:** Age sequence determined from the dating results of the opposite samples AZA-3 and AZA-13 collected from Anzapalivoro.

Sample/ segment code	Distance from the cavity wall (m)	Distance from the outer part/exterior (m)	Distance from the point of maximum age (m)	Age (yr)
-	0	1.45	0.52	0
AZA-3a	0.32	1.13	0.20	375
AZA-3b	0.42	1.03	0.10	465
AZA-3c	0.52	0.93	0	595
AZA-3d	0.62	0.83	0.10	470
AZA-3e	0.72	0.73	0.20	330
AZA-13b	0.73	0.72	0.21	325
AZA-13a	1.20	0.25	0.48	110
-	1.45	0	0.73	0

We also dated 2 segments from the second longest cavity sample AZA-2, which exhibit the same increase from the sampling point with the depth in the wood. In this case, as compared to the thickness of the cavity walls in this area (1.95 m), *i*.*e*., the distance between the opposite sampling points AZA-2 and AZA-12, the sample was too short (0.52 m) for reaching the point of maximum age which is positioned in a small hollow.

### Architectures of the investigated *A*. *za* specimens

The age sequence of the cavity samples demonstrates that Anzapalivoro possesses a closed ring-shaped structure with an accessible false cavity inside, identical to that identified by us previously for several large and old *A*. *digitata* specimens [11, 28].

All baobabs with ring-shaped structures are multi-stemmed. The stems describe at ground level a circle or an ellipse with an empty space inside. These stems are pointed upward and are fused almost perfectly, closing a false inner cavity. The ring-shaped structures are formed progressively and close over time, as they consist of stems which typically have different ages. The stems grow faster along the outer circumference and each develops a kind of crescent shape, which is necessary for fusing.

As we have mentioned, false cavities are natural empty spaces between the fused stems that build a closed ring-shaped structure. The thickness of the fused stems, that define the false cavity walls, is typically between 1.5 and 2.5 m. The oldest part of each stem is located between the false cavity walls and the outer part/exterior of the respective stem, always closer to the cavity, in an area which is accessible to the increment borer. Unlike normal cavities, formed by wood removal, which have usually irregular shapes and are not very tall, false cavities have more regular shapes, their lower part located at ground level, are larger, taller and covered by bark. The first noticeable difference between false and normal cavities is the presence or absence of the bark inside the cavity. While normal cavities become larger over time due to continuous decay, false cavities tend to become smaller due to stem growth [11, 28].

This general description of the closed ring-shaped structure with a false cavity inside, provided by us for *A*. *digitata*, fits perfectly for the biggest *A*. *za* specimen. An inspection of its trunk, fusion lines, canopy, cavity and especially the radiocarbon dating results, as will be shown further, indicate that Anzapalivoro consists of 5 fused stems. Thus, the quasi-complete ring is formed by 5 fused stems, which close a large and very tall false cavity. The width of the cavity walls vary between 1.40 and 2.10 m. The relatively small entrance to the false cavity will close probably over the next decades. Thus, *A*. *za* becomes the third *Adansonia* species, after *A*. *digitata* and *A*. *grandidieri* [11, 17, 28], which may exhibit a closed ring-shaped structure with a false cavity inside.

On the other hand, the Big cistern za has an obvious cluster structure, which is composed of 4 partially fused stems.

### Ages and growth of the investigated *A*. *za* specimens

For Anzapalivoro, the analysis of the oldest dated segments of the 3 cavity samples allows us to estimate the ages of the corresponding stems, which are identical to the so-called points of maximum age in the sampling direction (located between the cavity walls and the outer part). Given that the measured or extrapolated ages in the points of maximum ages of the cavity samples are different, we conclude that the 3 samples originate from 3 different stems, which were labelled I, II and III (Fig 7).

For the longest cavity sample AZA-3 (0.72 m), which is a special case, we found that the point of maximum age is located very close to the original position of segment AZA-3c (0.52 m). This segment was dated to 557 ± 19 bp, which corresponds to a calibrated age of 595 ± 10 yr. According to these results, the age of stem III should be close to 600–650 yr.

In the sampling point AZA-3, which is opposite to AZA-13, the width of the cavity wall, *i*.*e*., the distance from the cavity wall to the outer part, is 1.45 m. Thus, the point of maximum age is located at ca. 0.52 m from the cavity wall and ca. 0.93 m from the outer part. These values correspond to the following ratios for the point of maximum age (pma): distance from cavity wall to pma *vs*. distance from pma to outer part: 0.56; distance from cavity wall to pma *vs*. width of cavity wall (distance from cavity wall to outer part): 0.36; distance from pma to outer part *vs*. width of cavity wall: 0.64.

For the cavity sample AZA-2 (0.51 m), the innermost/deepest segment AZA-2b (0.51 m) is the oldest dated segment of Anzapalivoro. Its radiocarbon date of 780 ± 30 bp corresponds to a calibrated age of 735 ± 15 yr. In the sampling point AZA-2, which is opposite to AZA-12, the width of the cavity wall is 2.00 m. If we use the ratios found for sample AZA-3, the point of maximum age must be positioned at a distance of ca. 0.72 m from the cavity wall and 1.28 m from the outer part. In a conservative estimate, by taking into account growth rates and the decrease of growth with age, the age of stem II from which sample AZA-2 originates is close to 850 yr. The fact that the sample length was of only 0.51 m, even if the penetration of the increment borer was quasi-complete, indicates that after this distance there is a hollow in the cavity wall due to decay, which also includes the area of the point of maximum age.

The innermost segment of the shortest sample AZA-1 (0.31 m), *i*.*e*., AZA-1a, was radiocarbon dated to 440 ± 20 bp, which corresponds to a calibrated age of 545 ± 20 yr. The width of the cavity wall in the sampling point AZA-1 is 1.85 m. By using the same ratios, the point of maximum age is located at 0.67 m from the cavity wall and 1.18 m from the outer part. According to these values and considering the age of segment AZA-1a as well, we estimate that in the point of maximum age, which is missing due to another hollow in the cavity wall, the age of stem I is around 850–950 yr, *i*.*e*., 900 ± 50 yr.

Anzapalivoro has two more stems, that define the two sides of the cavity corridor, which were labelled IV and V (Fig 7). The ring is not yet completely closed, which allows for the presence of the cavity entrance and of the corridor. Due to the low width and height of the corridor, it was not possible to handle the increment borer for collecting samples from these stems. However, it is very probable that stems IV and V are the youngest ones. In a rough estimate, their ages would be around 500 yr. One should also mention that both the cavity entrance and the corridor will close over several decades; therefore, the ring will be completely closed.

According to the dating results and their extrapolation in the points of maximum age, the oldest part of the tree corresponds to stem I and would have an age of 900 ± 50 yr. According to this value, Anzapalivoro has started growing around ad 1115.

The rounded estimated ages of stems I, II, III, IV and V, namely 900, 850, 625, 500 and 500 yr, allow to estimate the way the ring was formed. The oldest stem I, followed immediately by stem II, represent the starting area of the ring. The ring closed progressively anti-clockwise (I → III → IV) and clockwise (II → V). The 5 stems of Anzapalivoro belong to three different generations.

We also dated the outermost segments of samples AZA-2 and AZA-12, which were adjacent to the cavity bark and to the bark of the outer part. The dating results provide negative radiocarbon dates, which are difficult to be interpreted and implies the use of (Post)Bomb calibration curves that yield very young calibrated ages. That is why these results have not been included in Table 1. They suggest that even the oldest stems of Anzapalivoro continue their growth both toward the cavity and toward the outer part. Taking into account all described aspects, we consider that the upper age limit of the *A*. *za* species is over 1,000 yr.

As previously noted, Anzapalivoro has a special architecture with a high symmetry, namely a closed ring-shaped structure, which is very suitable for baobabs to reach old ages. The different stems which build this structure grow from an area of maximum age (which consists of all points of maximum age) toward the interior and the exterior, building over time a closed or quasi-closed ring with a false cavity inside. By using the ages of segments extracted from the opposite samples AZA-3 and AZA-13, which were both collected at the height of 1.44 m above ground, and the distances presented in Table 2, one can determine the growth rates from the point of maximum age toward the interior (false cavity) and the exterior (outer part of the stem). Thus, stem III grew from the point of maximum age (AZA-3c) toward the interior by 0.10 m in the first 130 yr, by 0.10 m over the next 90 yr and by 0.32 m over the last 375 yr. Stem III grew simultaneously from the point of maximum age toward the exterior by 0.10 m during the first 125 yr, by 0.10 m over the next 140 yr, then by 0.38 m over another 230 yr and finally by 0.25 m over the last 110 yr. Because we neglected the age errors, these values must be considered as rather approximate.

The corresponding growth rates from the point of maximum age varied between 0.43–0.77 x 10^−3^ m yr^-1^ toward the interior and between 0.71–2.27 x 10^−3^ m yr^-1^ toward the exterior. One can also observe that stem III of Anzapalivoro started growing from an area of maximum age with comparable rates toward the interior and the exterior over the first 125–130 yr, after which the growth rates exhibited a decrease toward the interior and a considerable increase toward the exterior.

We already stated that the identification of complex and very unusual architectures of old baobabs, such as the closed ring-shaped structures with false cavities, the determination of stem ages and growth rates, mandatory requires the use of AMS radiocarbon dating of wood samples collected from determined positions. In such cases, ring counting and ring width analysis are not effective. According to our research, in the case of baobabs with closed ring-shaped structures, the number of rings between two dated segments is typically lower than the calendar age determined by radiocarbon dating. Usually, this difference increases with the age of the tree, *i*.*e*., for the wood produced when the baobab is already old. In addition, one should consider the existence of hollow parts and the ultimate growth stop phenomenon, identified by us for old stems [11, 16, 17]. In the case of Anzapalivoro, the sample AZA-3, from which we dated 5 tiny segments, can be divided into 5 smaller sample parts, which are delimited by the dated segments. For a given sample part, the ratio of counted rings *vs*. calendar years, calculated from radiocarbon dates, varies from 0.52 to 0.70.

The age of the Big cistern za can be determined from the dated segment of sample BCZ, which originates from the largest stem which includes the cistern. The diametre of this stem in the sampling direction, at sampling height (2.70 m), is of 2.80 m. The sampling point in the hollow part is only 0.20 m away from the position of the calculated pith, which corresponds to the dated segment. The calibrated age of segment BCZ, *i*.*e*., 260 ± 15 yr, indicates that the oldest stem of the Big cistern za is close to 260 yr.

## Conclusions

The research presents the results of the AMS radiocarbon investigation of Anzapalivoro, the largest known *A*. *za* specimen for establishing its architecture, growth and age. In addition, we investigated the Big cistern za, a large baobab with a human-made hollow used for water storage. The two representative za baobabs are located in Southwestern Madagascar.

For Anzapalivoro, two pairs of wood samples were collected from the inner cavity walls and from the outer part/exterior of the tree, in opposite directions. One additional sample was collected from the large inner cavity. Several segments extracted from these samples at determined distances were radiocarbon dated. For the cavity samples, dating results demonstrate a continuous increase of ages with the distance into the wood up to a point of maximum age, after which the values decrease toward the outer part. In prior work we identified this major anomaly in age sequences of cavity samples for large and old *A*. *digitata* specimens and also for some large *A*. *grandidieri* trees. The only reasonable explanation for this age anomaly is that such inner cavities are in fact false cavities, *i*.*e*., natural empty spaces between several fused stems which are disposed in a ring-shaped structure. The radiocarbon date of the oldest segment/sample was found to be 780 ± 30 bp, which corresponds to a calibrated age of around 735 yr. The dating results and the investigation of Anzapalivoro indicate that it consists of 5 perfectly fused stems, which build a closed ring-shaped structure with a false cavity inside. These stems belong to three different generations which are between 500 and 900 yr old. The oldest part of Anzapalivoro, which has a calculated age of 900 ± 50 yr, started growing around ad 1115. According to age sequences of samples, each stem started growing from an area of maximum age with comparable rates toward the interior and the exterior, after which the growth rates underwent a decrease toward the interior and a consistent increase toward the exterior.

The dating results indicate that the age of the second investigated specimen, the Big cistern za, which consists of 4 partially fused stems, is close to 260 yr.

The investigation of Anzapalivoro, which has a special architecture with a high symmetry, suggests that the upper age limit of the *A*. *za* species may be over 1,000 yr. Our research indicates, however, that the oldest trees of Madagascar belong to other baobab species, namely *A*. *rubrostipa* and *A*. *grandidieri*.
